# Signaling abnormality leading to excessive/aberrant synaptic plasticity in Alzheimer's disease

**DOI:** 10.3389/fnagi.2022.1062519

**Published:** 2022-10-31

**Authors:** Shigeki Kawabata

**Affiliations:** Dementia Project Promotion Office, Sompo Care Inc., Tokyo, Japan

**Keywords:** Alzheimer's disease, synapse, plasticity, amyloid beta precursor protein, presenilin, CTF, Rab5, SORL1

## Introduction

Alzheimer's disease (AD) is a progressive neurodegenerative disorder, pathologically characterized by the existence of amyloid plaques (APs) and neurofibrillary tangles (NFTs). The main constituent of APs is amyloid-β peptide (Aβ), whereas NFTs are aggregates of hyperphosphorylated tau protein. Early-onset familial Alzheimer's disease (FAD) pathologically and clinically bears a resemblance to the more common late-onset sporadic form of the disease (SAD). FAD is associated with defined mutations in amyloid β precursor protein (APP) and presenilin (PSEN1 and PSEN2). On the other hand, some SAD risk genes have been identified; one of the most well-studied is apolipoprotein E (APOE). APOE has three isoforms, APOE ε2, 3, and 4, and individuals with APOE ε4 are more likely to develop AD.

Synaptic plasticity describes the biological process that enables compensation for neuronal damage as well as learning and memory, through the remodeling of synaptic structures. Recently, it is proposed that excessive or aberrant synaptic plasticity is the pathophysiological basis for AD (Kawabata, 2022). APP is a key molecule for synaptic remodeling processes such as neurite extension and synaptogenesis (Müller et al., 2017). Presenilin modulates APP function as the catalytic core of γ-secretase complex, a protease complex responsible for APP processing (Zhou et al., 2017). It has been reported that APOE is also involved in synaptic plasticity through regulating APP function or metabolism (Huang et al., 2017, 2019; Lin et al., 2018). It is proposed that the function of APP is altered in FAD-linked mutant APP and presenilin, and that aberrant plasticity induced by such functional abnormality is a cause of FAD. With regards to SAD, although synaptic plasticity is basically a compensatory mechanism for overcoming age-related functional decline, errors arise during this process and synaptic plasticity occurs excessively (Grady, 2012). APOE ε4 has the strongest plasticity-promoting effect (Huang et al., 2017, 2019; Lin et al., 2018). In aging individuals with this risk factor, synaptic plasticity may tend to become excessive, and the risk of SAD may increase. It is hypothesized that a perturbation of activities associated with such excessive/aberrant synaptic plasticity lies at the heart of AD pathogenesis (Kawabata, 2022).

In the previous paper, however, the mechanism of APP signaling leading to excessive/aberrant synaptic plasticity was not discussed in detail. Through the probing of recent findings, which are primarily utilized to elucidate the defective intracellular trafficking as a neurodegenerative mechanism in AD (Kim et al., 2016; Xu et al., 2016; Kwart et al., 2019; Knupp et al., 2020; Hung et al., 2021; Mishra et al., 2022), here I explain that APP signaling in endosomes is critical for its remodeling-related function. I also discuss the possibility that APP signaling abnormality in endosomes causes excessive/aberrant synaptic plasticity, which can be a pathophysiological basis for AD.

## Processing and signal transduction of APP in endosomes

Proteolytic processing of APP occurs *via* two alternative pathways, localized to different subcellular compartments. Within the non-amyloidogenic α-pathway, the first step of the proteolysis is performed at the cell surface by α-secretase. It cleaves APP within Aβ sequences and produces secreted sAPPα and the membrane anchored C-terminal fragment (CTF) called CTFα (Koo and Squazzo, 1994). In the amyloidogenic β-pathway, β-secretase [the β-site APP cleaving enzyme 1 (BACE1)] cleaves APP in endosomes, which results in the generation of sAPPβ and CTFβ (Winckler et al., 2018). Subsequently, CTFα and CTFβ are both cleaved by γ-secretase and Aβ is produced from CTFβ.

Endosomes are essential sites of signal transduction. Signals transmitted from endosomes are different from those that arise from the plasma membrane (Murphy et al., 2009). One of the key molecules for endosomal signaling is Ras-related protein in brain 5 (Rab5), a modulator of early endosomal formation, which upon activation, interacts with its effectors, transmits signals and promotes cell growth, migration, proliferation and motility (Mendoza et al., 2014; Yuan and Song, 2020). Interestingly, it has become evident that CTFβ but not CTFα can induce the activation of Rab5 (Kim et al., 2016; Xu et al., 2016). Rab5 is engaged in neurite outgrowth and guidance in neurons (Sann et al., 2009). It mediates distinct signaling depending on the cues. Brain Derived Neurotrophic Factor induces dendritic branching through activating Rab5 (Moya-Alvarado et al., 2018). On the contrary, Nerve Growth Factor (NGF) down-regulates Rab5 activity to facilitate neurite outgrowth in PC12 cells (Liu et al., 2007). During neurite outgrowth, APP is localized at the P-domain of growth cones, where it co-exists with Rab5 in synaptic organelles that are distinct from synaptic vesicles (Sabo et al., 2003). The P-domain of growth cones is enriched in dynamic actin filaments, which are essential for the formation and movement of growth cone lamellipodia and filopodia. The activation of Rab5 induces cytoskeletal reorganization and lamellipodia formation. It is suggested that endosomes containing both APP and Rab5 link cytoskeletal and membrane dynamics and guide growth cones toward their targets (Sabo et al., 2003).

**Figure 1:**
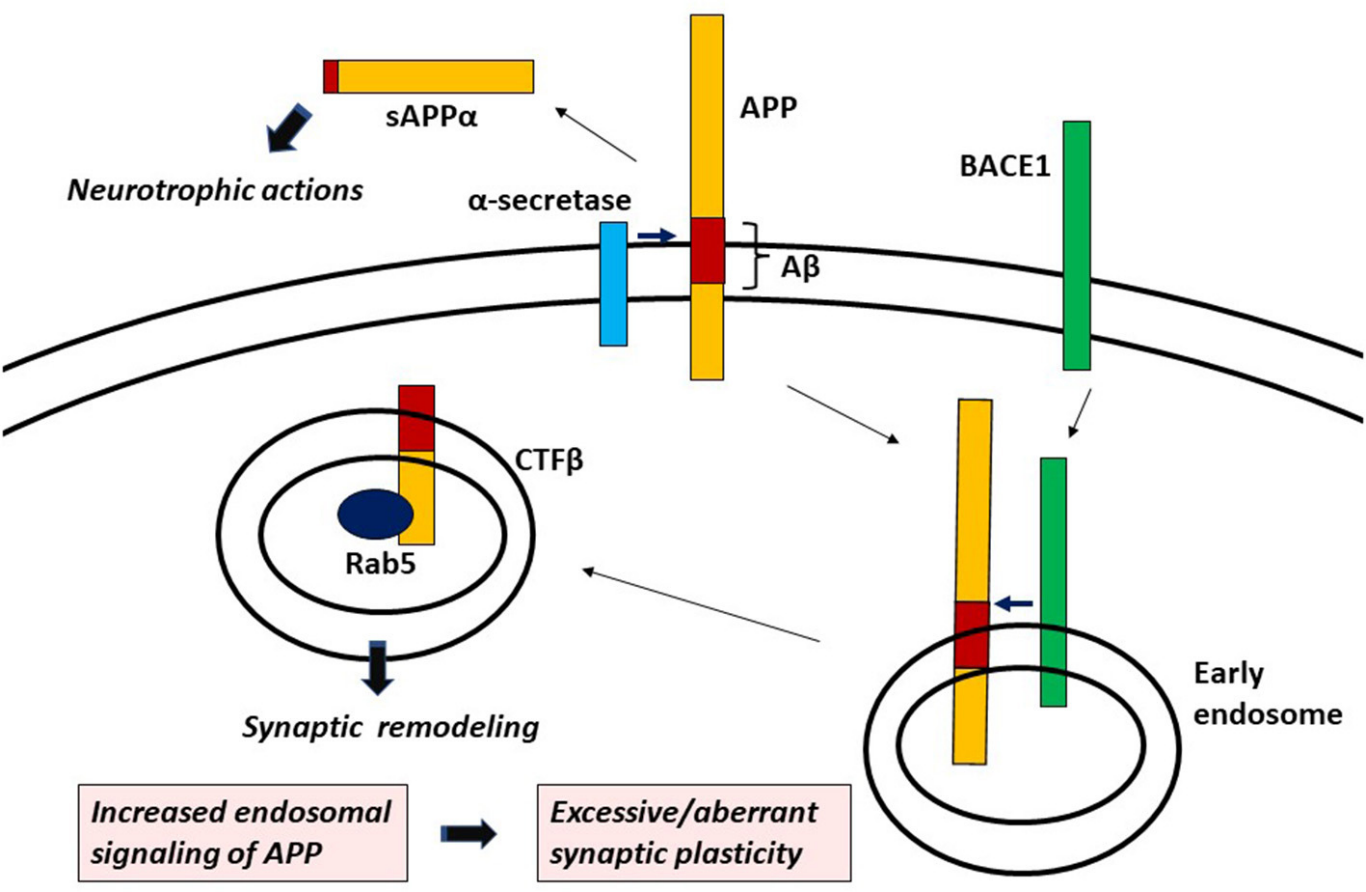
APP signaling leading to aberrant/excessive synaptic plasticity.

Although extracellularly released sAPPα is known to have neurotrophic actions (Müller et al., 2017), genetic studies in mice and *Drosophila* revealed that the CTF, especially the cytoplasmic domain of APP is responsible for its remodeling-related function (Torroja et al., 1999; Leyssen et al., 2005; Soldano et al., 2013; Klevanski et al., 2015). Altogether, endosomal signaling of APP, that is CTFβ (but not CTFα)-induced Rab5 activation seems to be involved in synaptic remodeling by APP (Figure 1). Interestingly, sAPPβ cannot exercise neurotrophic actions like sAPPα (Müller et al., 2017), indicating that the α-pathway or the β-pathway plays a role in extracellular or intracellular function of APP, respectively.

## Endosome/lysosome abnormalities in AD

A study using induced pluripotent stem cell (iPSC)-derived neurons carrying isogenic APP and PSEN1 mutants revealed that early endosomal enlargement is a common morphological phenotype observed among these neurons (Kwart et al., 2019). In cortical neurons from iPSC derived from individuals with FAD-linked mutant APP or PSEN1, impaired axonal lysosome transport and proteolysis deficits are seen (Hung and Livesey, 2018). Overexpression of APP in mouse cortical neurons markedly slow the velocity of axonal endosome transport (Kim et al., 2016), and CTFβ overexpressed in cultured rat basal forebrain cholinergic neurons inhibits retrograde axonal transport of NGF (Xu et al., 2016). The sortilin related receptor 1 (SORL1) encodes an endocytic receptor involved in protein trafficking between the trans-Golgi network and endosomes. Stop-gain and frameshift mutations of SORL1 are found to be causal to AD (Holstege et al., 2017; Hung et al., 2021). These mutations lead to the haploinsufficiency; the remaining copy of the gene alone is unable to preserve normal function. In neurons from iPSC derived from an individual with AD harboring the truncating mutation of SORL1, early endosomal enlargement is observed (Hung et al., 2021). These studies suggest that the defective intracellular trafficking is a pathological process associated with AD.

Nonetheless, endosomal CTFβ accumulation as well as Rab5 protein increase are also uniformly demonstrated in iPSC-derived neurons carrying various isogenic APP and PSEN1 mutations (Kwart et al., 2019). It is also revealed that Rab5 overactivation induces increased glycogen synthase kinase-3β (GSK-3β) activation and tau hyperphosphorylation in cortical neurons (Pensalfini et al., 2020), suggesting that exuberant APP signaling in endosomes deteriorates cytoskeletal organization leading to the accumulation of hyperphosphorylated tau, one of the pathological hallmarks of AD. Loss of SORL1 alters APP trafficking in endosomal networks, which results in increased localization of APP in early endosomes and decreased recycling to the plasma membrane (Knupp et al., 2020; Mishra et al., 2022). In neurons from iPSC derived from an individual with the truncating mutation of SORL1, an increase in Rab5 protein is observed (Hung et al., 2021). Collectively, it is suggested that SORL1 haploinsufficiency shifts the balance of APP processing toward the β-pathway, thereby increasing APP signaling in endosomes.

## Discussion

The appearance of pathological progression in AD is not a simple degenerative process. Network hypersynchrony occurs before the onset of clinical symptoms, which becomes hyposynchronous as the disease progresses. The default mode network (DMN), a resting-state network primarily composed of the medial prefrontal cortex, posterior cingulate cortex/precuneus and angular gyrus is hypersynchronous at a young age, which rather becomes hyposynchronous with aging in individuals carrying FAD-linked PSEN1 mutant (Quiroz et al., 2015; Badhwar et al., 2017) and in mice expressing FAD-linked mutant APP (Shah et al., 2018). The pattern of early DMN hypersynchrony and its hyposynchrony at a later age is similarly seen in APOE ε4 carriers when compared with non-carriers (Filippini et al., 2009; Su et al., 2015; Badhwar et al., 2017). Hippocampal hyperactivation during memory tasks is also common manifestation between young FAD-linked mutant carriers (Quiroz et al., 2010) and young APOE ε4 carriers (Filippini et al., 2009; Dennis et al., 2010). Again, as the disease progresses, hippocampus becomes hypoactive during memory tasks (Zott et al., 2018; Corriveau-Lecavalier et al., 2021).

This biphasic pattern of early network hypersynchrony and hyposynchrony at a later age is therefore an important feature linking FAD and SAD. Signaling abnormality which underlies such early network hypersynchrony may be an event trigger initiating the pathophysiological cascade of AD. Based on findings such as aberrantly increased excitatory synapses and dysregulated microcircuitry of GABAergic interneurons, which are primarily demonstrated in mice expressing FAD-linked mutant gene(s), in the previous paper, it is hypothesized that excessive/aberrant synaptic plasticity causes early network hypersynchrony seen before the clinical onset of AD (Kawabata, 2022).

In conclusion, CTFβ-induced endosomal signaling is responsible for synaptic remodeling by APP, which aberrantly increases with AD-related gene mutations. It can be proposed that increased APP signaling in endosomes such as Rab5 overactivation is the signaling mechanism leading to excessive/aberrant synaptic plasticity (Figure 1). Hyperactivation of APP/Rab5 signaling pathway can induce progressive neurodegeneration as well as tau hyperphosphorylation through GSK-3β activation (Pensalfini et al., 2020). Dystrophic neurites (DNs), which are typical constituents in APs, are known to form growth cones with abnormal extension of neurites (Masliah et al., 1991; Phinney et al., 1999). Noting that APP plays an important role in growth cone motility (Sabo et al., 2003), it is plausible that APP signaling abnormality leads to the formation of DNs through dysregulated axon guidance. It is suggested that abnormal signaling of APP not only builds early hypersynchronous neural network, but also triggers the process of neuritic degeneration. Hyperconnectivity of excitatory neural circuits causes progressive neuronal loss by glutamate excitotoxicity (Rogawski and Wenk, 2003), which may also account for neurodegeneration leading to hyposynchrony of neuronal network eventually seen at a later age. It is also known that synaptic remodeling, if excessive, causes maladaptive neuronal connectivity which deteriorates cognitive function (Grady, 2012). Although the defective intracellular trafficking mechanism may be involved in the process of neuritic degeneration in AD, excessive/aberrant synaptic plasticity caused by APP signaling abnormality in endosomes can be a root cause triggering the pathophysiological cascade of AD. Further elucidation of the mechanism of APP signaling in endosomes and its aberrancy in AD-related gene mutations or variants may fully unravel AD pathogenesis and hopefully help in the identification of novel drug targets.

## Author contributions

SK conceived the idea and wrote the paper.

## Conflict of interest

SK is employed by Sompo Care Inc.

## Publisher's note

All claims expressed in this article are solely those of the authors and do not necessarily represent those of their affiliated organizations, or those of the publisher, the editors and the reviewers. Any product that may be evaluated in this article, or claim that may be made by its manufacturer, is not guaranteed or endorsed by the publisher.
